# Supplementary material for: Clinico-Genomic Analysis Reveals Mutations Associated with COVID-19 Disease Severity: Possible Modulation by RNA Structure
Source: Pathogens. 2021 Aug 31;10(9):1109. doi: 10.3390/pathogens10091109 (PMC8464923; doi:10.3390/pathogens10091109)
Supplement: Supplementary file 1 [file pathogens-10-01109-s001.zip › pathogens-1283429-supplementary.pdf]

Mutation Association Analysis

Table S1: List of mutations associated with disease Severity

Mutation	Chi2	pValue	Pearson Correlation	Locus	Type of Mutation	Frequency	Severity Score
**26194|A|T**	9.308	0.0023	0.226	Orf3a	T268S	6.633	0.9231
**1373|C|T**	7.111	0.0077	-0.211	Orf1a	C370R	18.367	0.2778
26111|C|T	6.400	0.0114	-0.185	Orf3a	P240L	5.102	0.1000
**25611|C|A**	5.828	0.0158	0.187	Orf3a:74	synonymous	14.796	0.7241
**631|C|A**	5.121	0.0236	-0.177	Orf1a:122	synonymous	16.837	0.3030
**6312|C|A**	5.070	0.0243	-0.191	Orf1ab	T2016K	29.082	0.3509
10870|G|T	4.571	0.0325	-0.158	ORF1a:3535	synonymous	7.143	0.2143
**24622|T|C**	4.235	0.0396	-0.162	S:1020	synonymous	17.347	0.3235
1365|T|G	3.600	0.0578	-0.139	Orf1a	P371S	5.102	0.2000
15435|A|G	3.571	0.0588	-0.146	ORF1b:656	synonymous	14.286	0.3214
14408|C|T	2.456	0.1171	0.224	ORF1b	P314 L	75.000	0.5646
**28854|C|T**	2.380	0.1229	0.138	N	S194L	36.224	0.5915
241|C|T	2.320	0.1278	0.153	3'UTR	NA	49.490	0.5773
**3037|C|T**	2.273	0.1317	0.153	Orf1a:924	synonymous	50.510	0.5758
6633|C|T	2.273	0.1317	-0.111	ORF1a	A 2123 V	5.612	0.2727

Table S2: List of mutations associated with disease Mortality

Mutation	Chi2	pValue	Pearson Correlation	Locus	Type of Mutation	Frequency	Mortality Rate
**26194|A|T**	8.966	0.0028	0.221	Orf3a	T268S	6.633	0.3846
**6312|C|A**	5.482	0.0192	-0.199	orf1ab	T2016K	29.082	0.0175
**24622|T|C**	4.520	0.0335	-0.167	S:1020	synonymous	17.347	0.0000
**28854|C|T**	4.369	0.0366	0.187	N	S194L	36.224	0.1972
15772|A|T	4.054	0.0441	0.147	ORF1b	S 769 C	4.592	0.3333
1707|C|T	3.420	0.0644	-0.149	ORF1a	S481F	20.918	0.0244
**3037|C|T**	2.826	0.0928	0.171	Orf1a:924	synonymous	50.510	0.1717
18877|C|T	2.817	0.0933	0.177	Orf1b:1804	synonymous	54.082	0.1698

**1373|C|T**	2.788	0.0949	-0.132	Orf1a	C370R	18.367	0.0278
23929|C|T	2.654	0.1033	-0.134	S:789	synonymous	24.490	0.0417
12685|G|T	2.538	0.1111	-0.125	Orf1a	Q4140H	17.347	0.0294
**631|C|A**	2.414	0.1203	-0.122	Orf1a:122	synonymous	16.837	0.0303
2199|TTTA|T	2.393	0.1219	-0.116	Orf1a:644	deletion	9.184	0.0000
**25611|C|A**	2.245	0.1340	-0.116	Orf3a:74	synonymous	14.796	0.2069
1947|T|C	1.895	0.1686	-0.114	Orf1a	V561A	25.510	0.1800

Table S3: List of mutations associated with disease Severity (Co-morbidity)

**Mutation**	**Chi2**	**p Value**	**Pearson Correlation**	Locus	Type of Mutation	**Frequency**	**Severity Rate**
**26194|A|T**	5.571	0.018	0.237	Orf3a	T268S	9.174	1.000
10039|C|T	3.900	0.048	0.196	Orf1a:3258	synonymous	6.422	1.000
631|C|A	3.737	0.053	-0.193	Orf1a:122	synonymous	8.257	0.333
1373|C|T	3.737	0.053	-0.193	Orf1a	C370R	8.257	0.333
27870|G|T	3.343	0.067	0.180	Orf7b:39	E39*	5.505	1.000

Table S4: List of mutations associated with disease Mortality (Co-morbidity)

**Mutation**	**Chi2**	**p Value**	**Pearson Correlation Score**	Locus	Type of Mutation	**Frequency**	**Mortality Rate**
10870|G|T	4.880	0.027	0.218	orf1a	synonymous	5.607	0.500
6312|C|A	4.154	0.042	-0.217	orf1a	T2016K	19.626	0.000
**26194|A|T**	4.001	0.045	0.201	Orf3a	T268S	9.346	0.400
17115|T|C	2.556	0.110	0.159	Orf1b:1216	synonymous	7.477	0.375
12685|G|T	2.176	0.140	-0.149	Orf1a	Q4140H	10.280	0.000

Table S5: List of mutations associated with disease Severity (No Co-morbidity)

**Mutation**	**Chi2**	**p Value**	**Pearson Correlation Score**	**Locus**	**Type of Mutation**	**Frequency**	**Severity rate**

**11082|TG|T**	5.238	0.022	0.253	Orf1a:3606	L3606F	5.747	0.800
**11595|A|G**	5.238	0.022	0.253	Orf1a:3777	Q3777R	5.747	0.800
10870|G|T	3.797	0.051	-0.219	Orf1a:3535	synonymous	9.195	0.000
5884|C|T	3.360	0.067	0.201	Orf1a:1873	synonymous	4.598	0.750
15772|A|T	3.360	0.067	0.201	Orf1b:769	S769C	4.598	0.750

Table S6: List of mutations associated with disease Mortality (No Co-morbidity)

**Mutation**	**Chi2**	**p Value**	**Pearson Correlation Score**	**Locus**	**Type of Mutation**	**Frequency**	**Mortality rate**
**11082|TG|T**	10.830	0.001	0.363	Orf1a:3606	L3606F	5.747	0.429
15772|A|T	2.737	0.098	0.182	Orf1b:769	S769C	4.598	0.333
12312|C|T	2.737	0.098	0.182	Orf1a:4016	A4016V	4.598	0.250
**11595|A|G**	1.875	0.171	0.151	Orf1a:3777	Q3777R	5.747	0.176
27870|G|T	1.875	0.171	0.151	Orf7b:39	E39*	5.747	0.182

STRUCTURAL ANALYSIS

Protein structural analysis of Orf3a and N protein of SARS-CoV-2

We used the Innovagen's peptide calculator (https://pepcalc.com/) to study the effect of mutation on the N protein (Figure 1). It gives information about the molecular weight, net charge at neutral pH, information about solubility in water, and iso-electric point of the peptide. The site becomes polar after S is mutated to L at 194th site along with an increase in molecular weight.

A

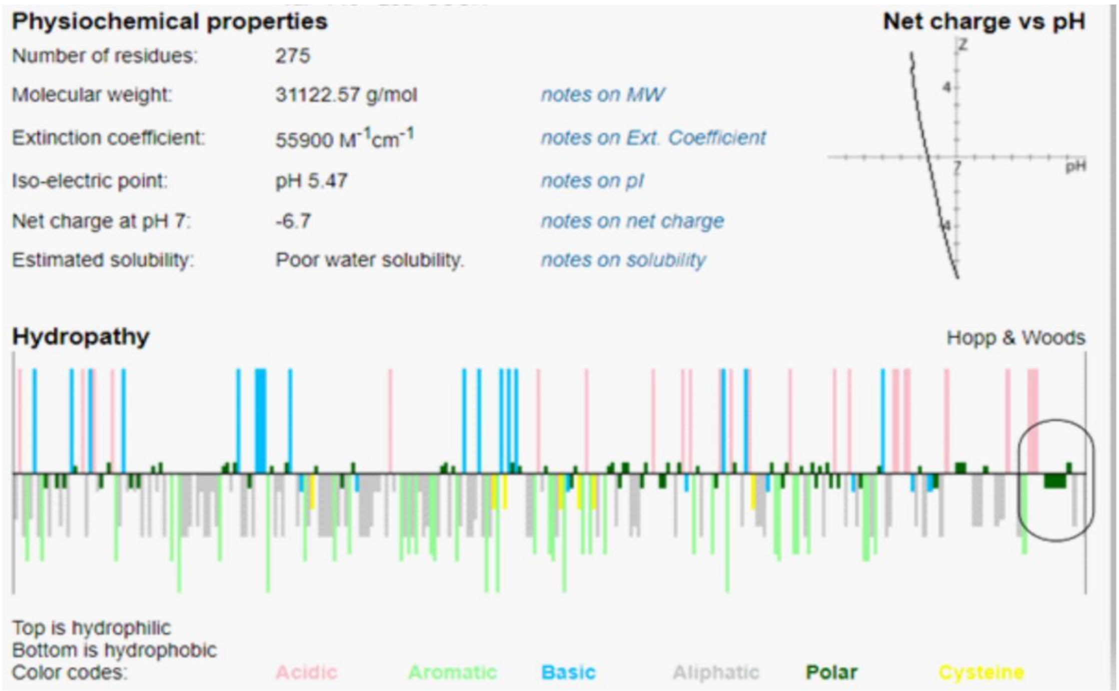

B

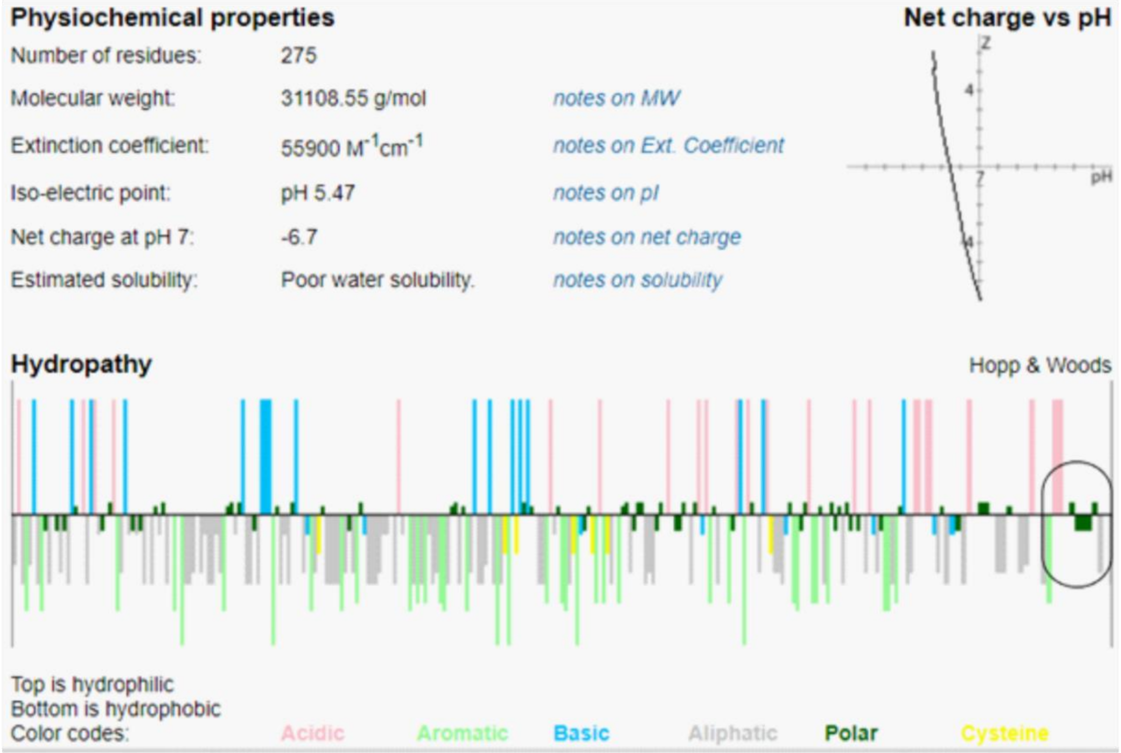

Figure S1: The various properties of residues of Orf3a. We used Innovagen's peptide calculator (https://pepcalc.com/) for the analysis of the S194L type of mutation associated with 28854 C|U in the N region and compared the effect on the physicochemical properties before**(a)** and after **(b)** the mutation.

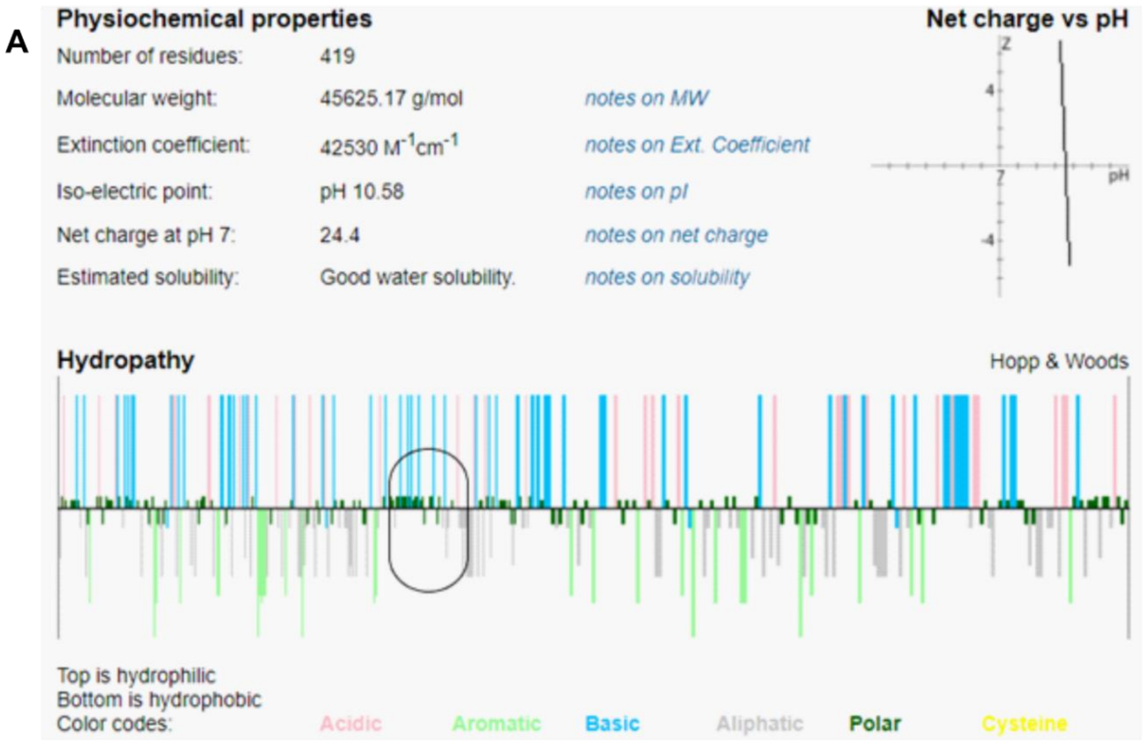

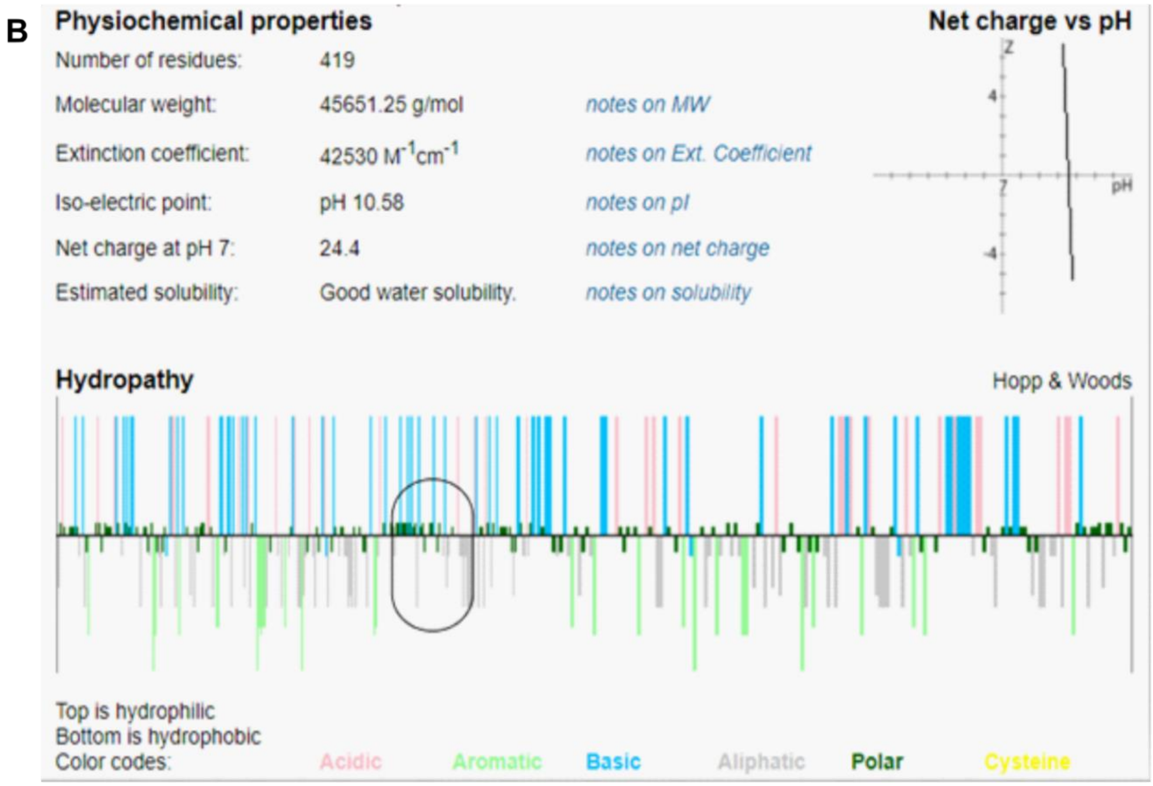

Figure S2: The various properties of residues of Orf3a for the T268S type of mutation associated with 26194 A|U in the Orf3a region and compared the effect on the physicochemical properties **(a)** before and **(b)** after the mutation.

Secondary structure of RNA and mutation

We analyzed the secondary structures of the RNA sequences using the RNAfold web server. We compared the effect of mutations on the secondary structure, by comparing the minimum free energy structure predicted by RNAfold before and after the mutation. The two mutations that we took into consideration are 25611 C|A in the Orf3a:74 locus, 28854 C|U in the N region. The 28854 C|U in the N region corresponds to the S194L type of mutation. The study revealed that both mutations led to the change in the secondary MFE structure (Figure 3 and 4).

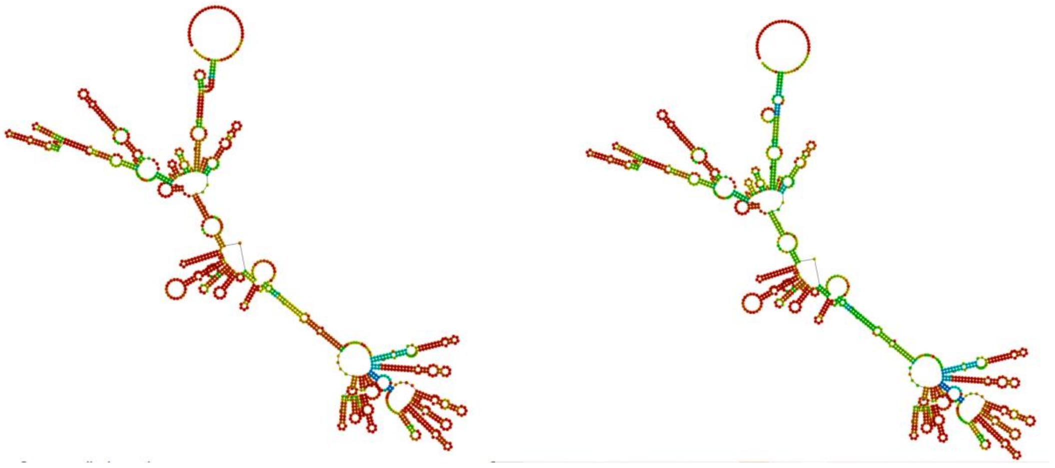

(a) (b)

Figure S3: Secondary Structure of N region (a) before mutation and (b) when C is mutated to T at 28854th site. Drastic change is observed in the RNA Secondary Structure after the mutation from C to U.

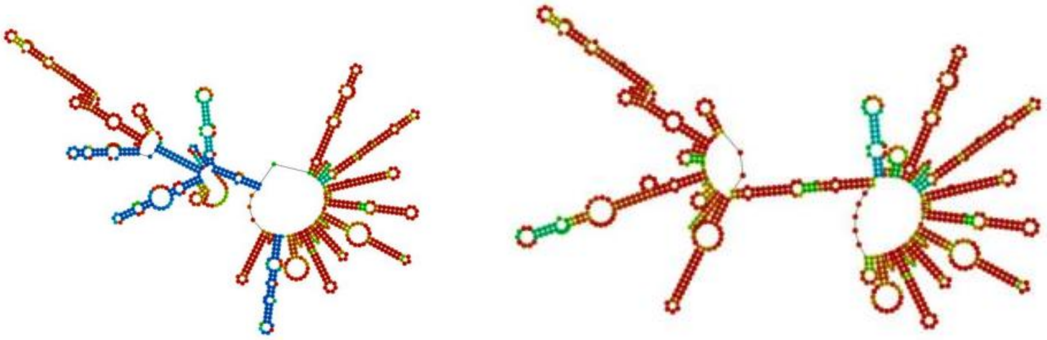

(a) (b)

Figure S4: MFE secondary structure comparison before (a) and after (b) the mutation at site 25611 from C to A. It can be observed that a drastic change takes place in the secondary structure.
